# Sex Differences in Dose Escalation and Overdose Death during Chronic Opioid Therapy: A Population-Based Cohort Study

**DOI:** 10.1371/journal.pone.0134550

**Published:** 2015-08-20

**Authors:** Eric Kaplovitch, Tara Gomes, Ximena Camacho, Irfan A. Dhalla, Muhammad M. Mamdani, David N. Juurlink

**Affiliations:** 1 Department of Medicine, University of Toronto, Toronto, Ontario, Canada; 2 Chronic Disease and Pharmacotherapy Research Program, Institute for Clinical Evaluative Sciences, Toronto, Ontario, Canada; 3 Department of Pharmacy, University of Toronto, Toronto, Ontario, Canada; University of British Columbia, CANADA

## Abstract

**Background:**

The use of opioids for noncancer pain is widespread, and more than 16,000 die of opioid-related causes in the United States annually. The patients at greatest risk of death are those receiving high doses of opioids. Whether sex influences the risk of dose escalation or opioid-related mortality is unknown.

**Methods and Findings:**

We conducted a cohort study using healthcare records of 32,499 individuals aged 15 to 64 who commenced chronic opioid therapy for noncancer pain between April 1, 1997 and December 31, 2010 in Ontario, Canada. Patients were followed from their first opioid prescription until discontinuation of therapy, death from any cause or the end of the study period. Among patients receiving chronic opioid therapy, 589 (1.8%) escalated to high dose therapy and n = 59 (0.2%) died of opioid-related causes while on treatment. After multivariable adjustment, men were more likely than women to escalate to high-dose opioid therapy (adjusted hazard ratio 1.44; 95% confidence interval 1.21 to 1.70) and twice as likely to die of opioid-related causes (adjusted hazard ratio 2.04; 95% confidence interval 1.18 to 3.53). These associations were maintained in a secondary analysis of 285,520 individuals receiving any opioid regardless of the duration of therapy.

**Conclusions:**

Men are at higher risk than women for escalation to high-dose opioid therapy and death from opioid-related causes. Both outcomes were more common than anticipated.

## Introduction

Over the past twenty years, the use of opioids for chronic noncancer pain has increased markedly in North America [1,2,3,4,5], with patients often receiving doses far in excess of those originally suggested for treatment [6]. The increasing use of opioids has been paralleled by a dramatic rise in opioid-related mortality.[7,8,9] More than 16,000 people now die annually of opioid-related causes in the United States alone, and similar trends have emerged in Canada, Australia, and Europe [10]. Many of these deaths could be avoided by more judicious prescribing of opioids in patients with chronic pain [10,11,12].

To minimize the risk of addiction and its attendant harms, clinical practice guidelines advocate various decision tools to stratify individual risk before initiating opioid therapy [5,13]. However, subjectivity remains in many aspects of opioid therapy [5,13,14]. This is supported by the observation that social and demographic factors, such as age, race and socioeconomic status, influence opioid prescribing patterns [15,16,17].

Few studies have explored the role of biological sex as it pertains to the safe prescribing of opioids in patients with chronic pain. There are several reasons why opioids might be prescribed differently to men and women, including differences in pain perception [18] and drug-seeking behaviour[19], as well as physician bias in the assessment of pain and formulation of a treatment plan [20]. Previous research suggests that women are more likely to be prescribed opioids, but men tend to receive more potent agents [1,15,21,22]. However, the majority of these studies are descriptive and focus only on initial prescriptions. [1,15,21,22] One small cohort study [23] examined the relationship between sex and dose escalation but had limited statistical power and did not examine mortality.

We sought to formally explore the relationship between sex, dose escalation and death due to overdose in a large cohort of patients receiving chronic opioid therapy for noncancer pain.

## Methods

### Setting

We performed a population-based cohort study among Ontarians between 15 and 64 years of age who received publicly funded opioid prescriptions between April 1^st^ 1997 and December 31^st^ 2010. These individuals have universal access to prescription drug coverage, hospital care and physicians’ services. All analyses were performed at the Institute for Clinical Evaluative Sciences (ICES), where various healthcare administrative databases are linked and analyzed in an anonymous fashion using encrypted, 10-digit health card numbers. This project was approved by the Research Ethics Board of Sunnybrook Health Sciences Centre, Toronto, Canada.

### Data Sources

We identified prescription records using the Ontario Drug Benefit Database, which contains comprehensive and highly accurate data of prescriptions dispensed to Ontarians eligible for public drug coverage [24]. Eligibility criteria for drug coverage among people younger than 65 years include disability, receipt of social assistance, high prescription drug costs relative to net household income, receipt of home care services and residence in a long-term care facility. We obtained demographic information from the Registered Persons Database, which contains one record for every Ontarian issued a health card number. We used the Ontario Cancer Registry to identify any previous diagnosis of cancer[25]. We used the Ontario Diabetes Database to ascertain the presence of diabetes [26], for inclusion in the Charlson Comorbidity Index. Inpatient hospitalization records were identified from the Canadian Institute for Health Information’s Discharge Abstract Database, and physician billing claims were obtained from the Ontario Health Insurance Plan Database.

Opioid-related deaths were identified by manual abstraction of records from the Office of the Chief Coroner for Ontario, as done previously [7,27]. These data are complete until December 31, 2011. By law, all deaths that do not arise from natural causes or are sudden and unexpected must be reported to the coroner. For this study, opioid-related deaths were defined as those in which the coroner’s investigation concluded so, based either on postmortem toxicology revealing opioid concentrations sufficiently high to cause death, or the coroner’s determination that a combination of drugs, including at least one opioid present at a clinically significant concentration, resulted in death. Deaths were considered unrelated to opioid use if another drug was present at a concentration high enough to cause death, even when one or more opioids were detected at levels that could be associated with therapeutic use[7].

### Study Design

We identified cohorts of men and women who commenced treatment with an opioid, based upon prescriptions for oral codeine, morphine, oxycodone, hydromorphone or transdermal fentanyl. We restricted the analysis to patients newly treated with opioids by excluding those with any other opioid prescription in the preceding year. We did not include prescriptions for hydrocodone, which is available in Canada only as a liquid antitussive, or for methadone, which is almost exclusively prescribed for opioid addiction rather than for pain in Ontario.

Each patient’s observation began on the date of their first opioid prescription. Patients were followed from their first opioid prescription until discontinuation of opioid treatment (defined as an interval of more than 120 days between successive prescriptions), death from any cause, or the end of study period (December 31^st^, 2010), whichever occurred first. The maximum duration of publically-funded prescriptions in Ontario is 100 days. For patients with more than one eligible cohort entry date (by virtue of intervals greater than 120 days between successive prescriptions), we studied only the first course of therapy. To restrict the analysis to patients with noncancer pain, we excluded from the analysis individuals with any evidence of cancer prior to cohort entry, as well as patients with any physician claim or inpatient hospitalization for palliative care services in the 180 days preceding cohort entry.

In the primary analysis we studied patients receiving chronic opioid therapy, which we defined as three or more months of opioid treatment. These patients had one or more opioid prescriptions at least 91 days following their first prescription, with no interval of 120 days or more between successive prescriptions. In a secondary analysis, we examined all patients who commenced opioid therapy, regardless of the overall duration of treatment. While most such prescriptions represent short-term treatment for pain and do not progress to chronic therapy, we conducted this analysis because dose escalation and opioid-related death do sometimes occur in the first 3 months of therapy.

The primary outcome was defined as escalation to a daily opioid dose of more than 200 milligrams of morphine or equivalent. We chose this dose because it has been identified as a threshold in both American [5] and Canadian guidelines [13] and because higher doses confer increasing risk of adverse outcomes while lower doses are sufficient for pain control in the vast majority of patients. Our previous work demonstrates that doses exceeding 200 mg of morphine or equivalent increase the risk of motor vehicle collisions [28] and opioid-related mortality [27].

Dose was ascertained as described previously [27,28], incorporating the strength and number of tablets dispensed, the days supplied by the prescription and the potency of the opioid relative to morphine, defined using equivalence ratios published by the National Opioid Use Guideline Group [13]. The secondary outcome examined death from opioid-related causes, defined using provincial coroner’s data.

### Statistical Analysis

We used standardized differences to compare baseline characteristics of men and women who commenced opioid therapy. Unlike significance testing, standardized differences are not influenced by sample size; values lower than 0.10 suggest negligible differences in the mean value of the characteristic between groups [29]. We therefore used Cox proportional hazards regression to estimate the risks of dose escalation and opioid-related mortality for men relative to women, after adjusting for all variables with a standardized difference greater or equal to 0.10, including age, documented alcohol-related disorder in the preceding 5 years, number of distinct non-opioid medications prescribed in the past 6 months and use of serotonergic antidepressants. We also adjusted for receipt of benzodiazepines, antipsychotic drugs and other psychotropic drugs or central nervous system (CNS) depressants in the preceding 180 days, as well as comorbidity defined using the Charlson comorbidity index [30].

The proportional hazards assumption was verified using a time-dependent exposure covariate and by inspection of log-log survival curves. We constructed Kaplan-Meier curves to characterize the incidence of both outcomes over time. All analyses were performed with SAS version 9.2 (SAS institute Cary N.C.) and used a two-tailed type I error rate of 0.05 as the threshold for statistical significance.

## Results

During the 13-year study period, we identified 285,520 individuals who commenced treatment with an opioid. Within this cohort, 32,449 (11.4%) continued opioid therapy for 3 months or more, including 13,640 (42.0%) men and 18,809 (58.0%) women. Overall, 589 patients (1.8%) escalated to high dose therapy over a median follow-up of 186 days (interquartile range 117 to 442 days), while 59 patients (0.2%) died of opioid-related causes at a median of 2.6 years (interquartile range 1.1 to 5.2 years) from their first opioid prescription.

Compared to women, men receiving opioids were slightly older, more likely to have a documented alcohol use disorder, and less likely to receive antidepressants (Table 1). Men and women were otherwise similar with regard to demographics, comorbidity measures, physician utilization and medication use at baseline.

**Table 1:** Baseline characteristics of men and women receiving chronic opioid therapy.

Characteristic		Men N = 13,640	Women N = 18,809	Standardized Difference
Age (median, IQR)		47 (38–56)	44 (33–55)	0.23
Documented alcohol use disorder (5 years)		2,262 (16.6%)	1,243 (6.6%)	0.33
Past medication use (180 days)				
	SSRIs/SNRIs	2,531 (18.6%)	4,592 (24.4%)	0.14
	Other antidepressants	1,788 (13.1%)	2,868 (15.2%)	0.06
	Benzodiazepines	3,440 (25.2%)	4,925 (26.2%)	0.02
	Antipsychotics	1,573 (11.5%)	1,643 (8.7%)	0.09
	Other psychotropic drugs & CNS depressants	432 (3.2%)	627 (3.3%)	0.01
Charlson Score				
	No hospitalizations	8,011 (58.7%)	9,808 (52.1%)	0.08
	0	2,781 (20.4%)	6,491 (34.5%)	0.08
	1	1,256 (9.2%)	1,481 (7.9%)	0.08
	≥2	1,592 (11.7%)	1,029 (5.5%)	0.08
Physician visits in past year (median IQR)		15 (8–27)	16 (9–28)	0.01
Median number of distinct drugs used in past 6 months (IQR)		4 (2–8)	5 (3–8)	0.14
Neighborhood income quintile				
	1	5,782 (42.4%)	8,312 (44.2%)	0.06
	2	3,086 (22.6%)	4,355 (23.2%)	0.06
	3	2,003 (14.7%)	2,736 (14.5%)	0.06
	4	1,535 (11.3%)	1,951 (10.4%)	0.06
	5	1,152 (8.4%)	1,378 (7.3%)	0.06
	Missing information	82 (0.6%)	77 (0.4%)	0.06
Residence				
	Urban	11,615 (85.2%)	16,266 (86.5%)	0.04
	Rural	1,976 (14.5%)	2,507 (13.3%)	0.04
	Missing information	49 (0.4%)	36 (0.2%)	0.04
Initial opioid				
	Immediate release combination opioids	12,682 (93.0%)	17,515 (93.1%)	0.03
	Immediate release single-agent opioids	507 (3.7%)	741 (3.9%)	0.03
	Long acting morphine	186 (1.4%)	213 (1.1%)	0.03
	Long acting oxycodone	117 (0.9%)	136 (0.7%)	0.03
	Transdermal fentanyl	67 (0.5%)	117 (0.6%)	0.03
	Long acting hydromorphone	46 (0.3%)	55 (0.3%)	0.03
	Long acting codeine	35 (0.3%)	32 (0.2%)	0.03

Abbreviations; IQR, interquartile range; SSRI, selective serotonin reuptake inhibitor; SNRI, serotonin-norepinephrine reuptake inhibitor; CNS, central nervous system

**Fig 1:**
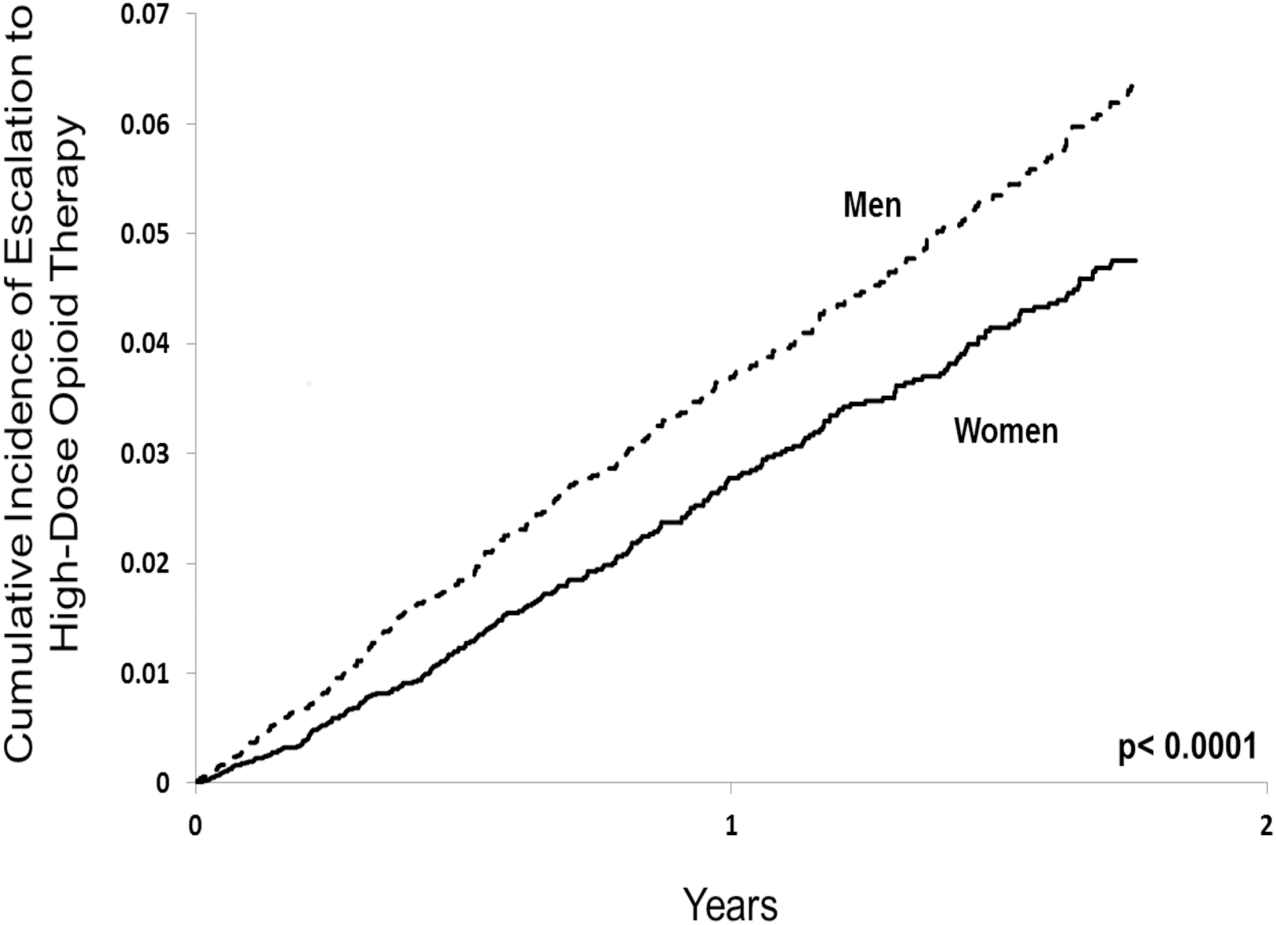
Escalation to high dose opioid therapy among men and women. Kaplan-Meier curves of opioid dose escalation to an average daily dose exceeding 200 mg of morphine (or equivalent) among men and women with at least 91 days of continuous opioid therapy. P values were determined by Cox proportional hazards regression.

**Fig 2:**
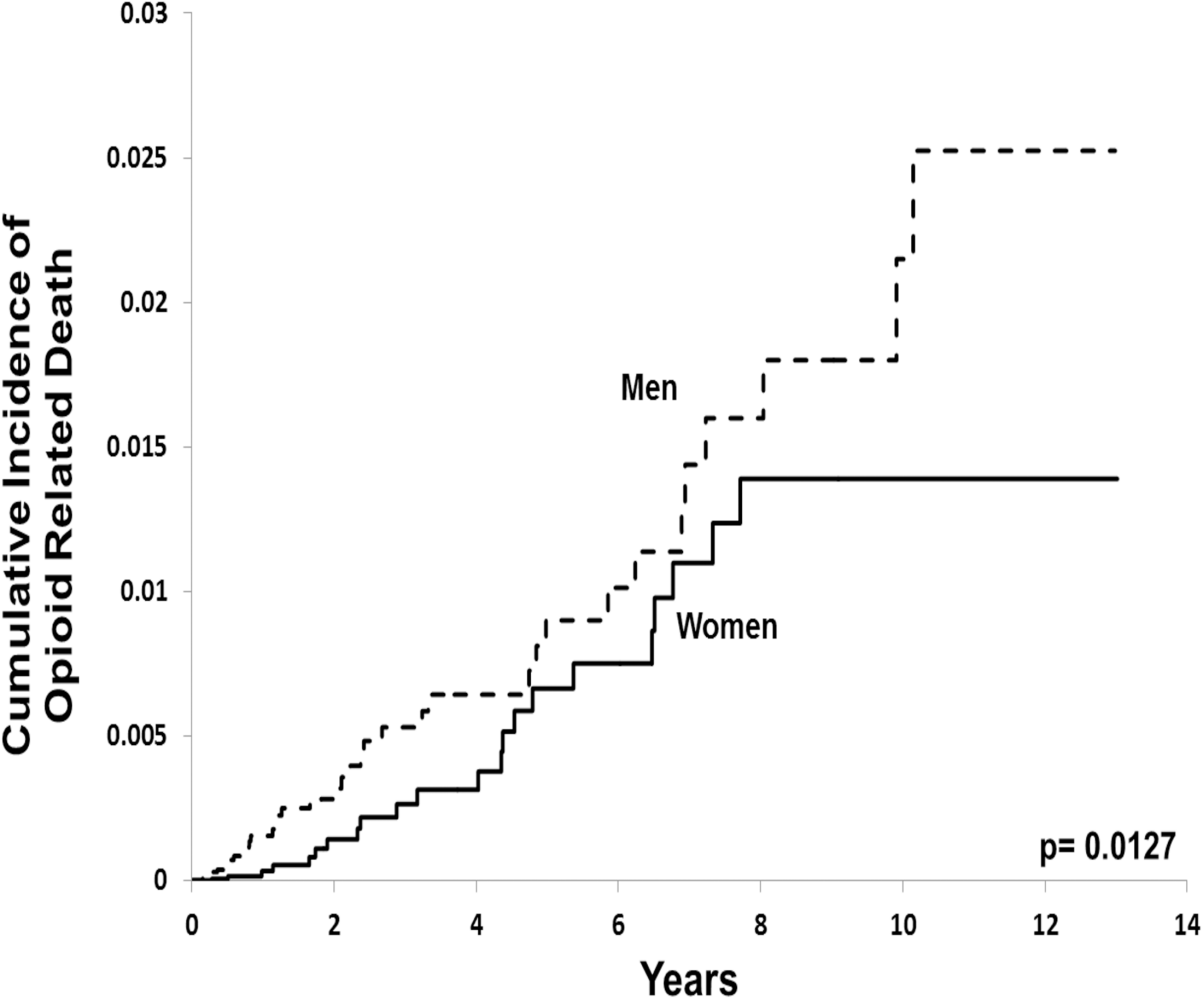
Opioid-related death among men and women. Kaplan-Meier curves of opioid-related mortality among men and women with at least 91 days of continuous opioid therapy. P values were determined by Cox proportional hazards regression.

Among patients receiving chronic opioid therapy, 319 (2.3%) men and 270 (1.4%) women escalated to high dose therapy, representing roughly 1 out of every 45 men and 1 out of every 70 women in our sample. In total, 37 (0.3%) men and 22 (0.1%) women died of opioid-related causes, representing 1 out of every 350 men and 1 out of every 850 women receiving chronic opioid therapy, respectively. After multivariable adjustment, men were almost 50% more likely to escalate to high-dose opioids (adjusted hazard ratio 1.44; 95% confidence interval, 1.21 to 1.70; Table 2 and Fig 1) and twice as likely to experience an opioid-related death (adjusted hazard ratio 2.04; 95% confidence interval 1.18 to 3.53; Table 3 and Fig 2) compared to women. Those who escalated to high dose opioid therapy were nearly 24 times as likely to die as those who did not escalate (3.1% vs. 0.1%, respectively).

**Table 2:** Escalation to high dose opioid therapy among men and women^a^.

	Events among men (n, %)	Events among women (n, %)	Unadjusted Hazard Ratio (95% CI)^b^	Adjusted Hazard Ratio (95% CI)^b^
Patients on Chronic Opioid Therapy	319 (2.3%)	270 (1.4%)	1.40 (1.19 to 1.65)	1.44 (1.21 to 1.70)^c^,^d^
All Patients Commencing Opioid Therapy	445 (0.4%)	369 (0.2%)	1.50 (1.31 to 1.73)	1.52 (1.32 to 1.76)^c^

^a^ Follow up censored at 2 years

^b^ Hazard ratios presented for men, with women as reference group

^c^ Adjusted for patient age, history of alcohol abuse or alcohol-related comorbidity, previous SSRI use, previous other antidepressant use, previous benzodiazepine use, previous antipsychotic use, previous use of other CNS depressants, and Charlson score

^d^ Adjusted for median number of distinct non-opioid drugs used in past 6 months

**Table 3:** Opioid-related death during chronic opioid therapy in men and women.

	Events in Men (n, %)	Events in Women (n, %)	Unadjusted Hazard Ratio (95% CI)^a^	Adjusted Hazard Ratio (95% CI)^s^
Patients on Chronic Opioid Therapy	37 (0.3%)	22 (0.1%)	1.96 (1.15 to 3.32)	2.04 (1.18 to 3.53)^b^,^c^
All Patients Commencing Opioid Therapy	56 (0.1%)	35 (0.03%)	2.10 (1.38 to 3.21)	2.18 (1.40 to 3.38)^b^

^a^ Hazard ratios presented for men, with women as reference group

^b^ Adjusted for patient age, history of alcohol abuse or alcohol-related comorbidity, previous SSRI use, previous other antidepressant use, previous benzodiazepine use, previous antipsychotic use, previous use of other CNS depressants, and Charlson score

^c^ Adjusted for median number of distinct non-opioid drugs used in past 6 months

We found similar results in the analysis of all 285,520 subjects who received any opioid prescription, regardless of the duration of therapy. Overall, 814 patients (0.3%) escalated to high-dose therapy and 91 patients (0.03%) died of opioid overdose. In this analysis, men also faced an increased risk of escalation to high-dose opioid therapy (adjusted hazard ratio 1.52; 95% confidence interval 1.32 to 1.76; Table 2) and opioid-related death (adjusted hazard ratio 2.18; 95% confidence interval 1.40 to 3.38; Table 3) compared to women.

## Discussion

In this population-based cohort study spanning 13 years, we found that men prescribed opioids for chronic noncancer pain were more likely than women to escalate to high-dose therapy and die of opioid overdose, even after adjustment for age, comorbidity, documented alcohol-related disorder and medication use. Moreover, both outcomes were more common than anticipated, with 1 of every 55 patients escalating to high-dose opioid therapy and 1 of every 550 dying of an opioid-related cause.

The importance of these findings is highlighted by the fact that tens of millions of patients worldwide receive chronic opioid therapy each year. Sales of opioids have quadrupled in the last decade[8], and more than 238 million opioid prescriptions were issued in the United States in 2011 alone, making them the third most widely prescribed drug class after lipid-lowering drugs and antidepressants[31].

Determining which patients are at risk of opioid dose escalation, addiction or opioid-related death is challenging, and clinical practice guidelines [5,13] advocate various decision tools to stratify individual risk before initiating or up-titrating opioid therapy. Despite these efforts, opioid deaths have continued to increase [9]. Opioid-related deaths represent more than 40% of all mortality from poisoning [9] and now outnumber deaths from alcoholic liver disease and HIV in North America[10].

Our findings underscore the importance of revisiting the indications for chronic opioid therapy, particularly at high doses. This is especially true in light of the paucity of good evidence regarding the safety and effectiveness of long term opioid therapy [14,32,33], as well as the lack of evidence demonstrating their superiority to other analgesics in treating chronic noncancer pain[14,34]. It is important to recognize that, as our findings indicate, more than 10% of patients treated anew with opioids progress to chronic therapy. Moreover, by identifying males to be at greater risk for escalation and opioid-related mortality, physicians can exercise particular caution when considering opioids for chronic noncancer pain in men.

Ultimately, further studies are required both to elucidate the role of opioids in chronic noncancer pain and to discern which patients can tolerate opioids safely.

Our study represents the first population-based study to document biological sex as a significant predictor of opioid dose escalation and opioid-related death among patients commencing chronic opioid therapy. However, some limitations of our work merit emphasis. The study’s findings derive from subjects receiving publicly funded prescription coverage and may not be generalizable to all patients. However, this is a particularly relevant population because social disadvantage is associated with higher rates of both opioid prescribing and opioid misuse [3,35]. Second, we cannot determine with confidence why men face an increased risk of dose escalation and death during chronic opioid therapy. Although a propensity for high risk behaviours surrounding opioids among men may partly explain our findings [36,37], the explanation is likely complex and multifactorial. Third, the nature of our data makes it difficult to ascertain the appropriateness of diagnoses and treatment as well as adherence to prescribed medication. Similarly, we have no reliable data on recreational drug use, save for alcohol use disorder. Additional research on the role of other illnesses, particularly mental health disorders, is needed to better identify those at particular risk of opioid-related harms. Finally, it merits note that no female deaths occurred in the final 6 years of study. This most likely represents depletion of susceptible individuals [38] and competing mortality from other causes of death.

Importantly, our methods necessarily underestimate both the amount of opioids consumed by patients and the number of deaths occurring during chronic therapy. We have no information about receipt of opioids from other sources, including illicit sources or prescriptions paid in cash or by other insurers. We purposefully excluded patients with long intervals between prescriptions, as well as those who received methadone. Considering the extent of diversion [39] and the high opioid-related mortality attributed to methadone [40], the true risks of opioid therapy are likely greater than our findings suggest.

In summary, in a large population of patients initiating opioid therapy for chronic noncancer pain, we found that men were more likely than women to escalate to high-dose opioid therapy and more than twice as likely to die of an opioid overdose. Moreover, both outcomes were much more common than anticipated. Although our findings underscore the importance of judicious opioid use in both men and women, they suggest that particular vigilance be applied to men, especially as doses begin to escalate.

## References

[1] Gomes T, Juurlink DN, Dhalla IA, Mailis-Gagnon A, Paterson JM, Mamdani MM. Trends in opioid use and dosing among socio-economically disadvantaged patients. Open Med 2011;5:e13–e22. 22046214 PMC3205807

[2] Caudill-Slosberg MA, Schwartz LM, Woloshin S. Office visits and analgesic prescriptions for musculoskeletal pain in US: 1980 vs. 2000. Pain 2004;109:514–9. 15157714 10.1016/j.pain.2004.03.006

[3] Sullivan MD, Edlund MJ, Fan MY, Devries A, Brennan Braden J, Martin BC. Trends in use of opioids for non-cancer pain conditions 2000–2005 in commercial and medicaid insurance plans: The TROUP study. Pain 2008;138:440–9. 10.1016/j.pain.2008.04.027 18547726 PMC2668925

[4] Boudreau D, Von Korff M, Rutter CM, Saunders K, Ray GT, Sullivan MD, et al Trends in long-term opioid therapy for chronic non-cancer pain. Pharmacoepidemiol Drug Saf 2009;18:1166–75. 10.1002/pds.1833 19718704 PMC3280087

[5] Chou R, Fanciullo GJ, Fine PG, Adler JA, Ballantyne JC, Davies P, et al Clinical guidelines for the use of chronic opioid therapy in chronic noncancer pain. J Pain 2009;10:113–30. 10.1016/j.jpain.2008.10.008 19187889 PMC4043401

[6] Portenoy RK, Foley KM. Chronic use of opioid analgesics in non-malignant pain: report of 38 cases. Pain 1986;25:171–86. 2873550 10.1016/0304-3959(86)90091-6

[7] Dhalla IA, Mamdani MM, Sivilotti ML, Kopp A, Qureshi O, Juurlink DN. Prescribing of opioid analgesics and related mortality before and after the introduction of long-acting oxycodone. CMAJ 2009;181:891–6. 10.1503/cmaj.090784 19969578 PMC2789126

[8] Centers for Disease Control and Prevention (CDC). Vital signs: Overdoses of prescription opioid pain relievers---united states, 1999–2008. MMWR Morb Mortal Wkly Rep. 2011;60:1487–92. 22048730

[9] Warner M, Chen LH, Makuc DM, Anderson RN, Minino AM. Drug poisoning deaths in the united states, 1980–2008. NCHS Data Brief 2011;81:1–8. 22617462

[10] Dhalla IA, Persaud N, Juurlink DN. Facing up to the prescription opioid crisis. BMJ 2011;343:d5142 10.1136/bmj.d5142 21862533

[11] Von KM, Kolodny A, Deyo RA, Chou R. Long-term opioid therapy reconsidered. Ann Intern Med 2011;155:325–8. 10.7326/0003-4819-155-5-201109060-00011 21893626 PMC3280085

[12] Nelson LS, Perrone J. Curbing the opioid epidemic in the United States: the risk evaluation and mitigation strategy (REMS). JAMA 2012;308:457–8. 10.1001/jama.2012.8165 22851109

[13] National Opioid Use Guideline Group (NOUGG). Canadian Guideline for Safe and Effective Use of Opioids for Chronic Non-Cancer Pain. 2010 Available: http://nationalpaincentre.mcmaster.ca/opioid/. Accessed 28 July 2013.

[14] Chou R, Ballantyne JC, Fanciullo GJ, Fine PG, Miaskowski C. Research gaps on use of opioids for chronic noncancer pain: findings from a review of the evidence for an American Pain Society and American Academy of Pain Medicine clinical practice guideline. J Pain 2009;10:147–59. 10.1016/j.jpain.2008.10.007 19187891

[15] Wisniewski AM, Purdy CH, Blondell RD. The epidemiologic association between opioid prescribing, non-medical use, and emergency department visits. J Addict Dis 2008;27:1–11. 10.1300/J069v27n01_01 18551883

[16] Joynt M, Train MK, Robbins BW, Halterman JS, Caiola E, Fortuna RJ. The Impact of Neighborhood Socioeconomic Status and Race on the Prescribing of Opioids in Emergency Departments Throughout the United States. J Gen Intern Med 2013. Epub ahead of print 2013 June 25. 10.1007/s11606-013-2516-z PMC3832731 23797920

[17] Pletcher MJ, Kertesz SG, Kohn MA, Gonzales R. Trends in opioid prescribing by race/ethnicity for patients seeking care in US emergency departments. JAMA 2008 2;299:70–8. 10.1001/jama.2007.64 18167408

[18] Fillingim RB, King CD, Ribeiro-Dasilva MC, Rahim-Williams B, Riley JL 3. Sex, gender, and pain: a review of recent clinical and experimental findings. J Pain 2009;10:447–85. 10.1016/j.jpain.2008.12.001 19411059 PMC2677686

[19] Back SE, Payne RL, Simpson AN, Brady KT. Gender and prescription opioids: findings from the National Survey on Drug Use and Health. Addict Behav 2010;35:1001–7. 10.1016/j.addbeh.2010.06.018 20598809 PMC2919630

[20] Green CR, Wheeler JR, LaPorte F. Clinical decision making in pain management: Contributions of physician and patient characteristics to variations in practice. J Pain 2003;4:29–39. 14622725 10.1054/jpai.2003.5

[21] Sadowski CA, Carrie AG, Grymonpre RE, Metge CJ, St John P. Access and intensity of use of prescription analgesics among older Manitobans. Can J Clin Pharmacol 2009;16:e322–e330. 19483264

[22] Campbell CI, Weisner C, Leresche L, Ray GT, Saunders K, Sullivan MD, et al Age and gender trends in long-term opioid analgesic use for noncancer pain. Am J Public Health 2010;100:2541–7. 10.2105/AJPH.2009.180646 20724688 PMC2978198

[23] Han H, Kass PH, Wilsey BL, Li CS. Age, gender, and earlier opioid requirement associations with the rate of dose escalation in long-term opioid therapy. J. Opioid Manag 2013;9:129–38 10.5055/jom.2012.0154 23709322

[24] Levy AR, O'Brien BJ, Sellors C, Grootendorst P, Willison D. Coding accuracy of administrative drug claims in the Ontario Drug Benefit database. Can J Clin Pharmacol 2003;10:67–71. 12879144

[25] Hall S, Schulze K, Groome P, Mackillop W, Holowaty E. Using cancer registry data for survival studies: the example of the Ontario Cancer Registry. J Clin Epidemiol 2006;59:67–76. 16360563 10.1016/j.jclinepi.2005.05.001

[26] Hux JE, Ivis F, Flintoft V, Bica A. Diabetes in Ontario: determination of prevalence and incidence using a validated administrative data algorithm. Diabetes Care 2002;25:512–6. 11874939 10.2337/diacare.25.3.512

[27] Gomes T, Mamdani MM, Dhalla IA, Paterson JM, Juurlink D.N. Opioid dose and drug-related mortality in patients with nonmalignant pain. Arch Intern Med 2011;171:686–91. 10.1001/archinternmed.2011.117 21482846

[28] Gomes T, Redelmeier DA, Juurlink DN, Dhalla IA, Camacho X, Mamdani MM. Opioid dose and risk of road trauma in Canada: a population-based study. JAMA Intern Med 2013;173:196–201. 10.1001/2013.jamainternmed.733 23318919

[29] Mamdani M, Sykora K, Li P, Normand SL, Streiner DL, Austin PC, et al Reader's guide to critical appraisal of cohort studies: 2. Assessing potential for confounding. BMJ 2005 23;330:960–2. 15845982 10.1136/bmj.330.7497.960 PMC556348

[30] Charlson ME, Pompei P, Ales KL, MacKenzie CR. A new method of classifying prognostic comorbidity in longitudinal studies: development and validation. J Chronic Dis 1987;40:373–83. 3558716 10.1016/0021-9681(87)90171-8

[31] IMS Institute for Healthcare Informatics. The Use of Medicines in the United States: Review of 2011. Available: http://www.imshealth.com/ims/Global/Content/Insights/IMS%20Institute%20for%20Healthcare%20Informatics/IHII_Medicines_in_U.S_Report_2011.pdf. Accessed 28 July 2013.

[32] Noble M, Tregear SJ, Treadwell JR, Schoelles K. Long-term opioid therapy for chronic noncancer pain: a systematic review and meta-analysis of efficacy and safety. J Pain Symptom Manage 2008;35:214–8. 10.1016/j.jpainsymman.2007.03.015 18178367

[33] Kissin I. Long-term opioid treatment of chronic nonmalignant pain: unproven efficacy and neglected safety? J Pain Res 2013;6:513–29. 10.2147/JPR.S47182 23874119 PMC3712997

[34] Furlan AD, Sandoval JA, Mailis-Gagnon A, Tunks E. Opioids for chronic noncancer pain: a meta-analysis of effectiveness and side effects. CMAJ 2006;174:1589–94. 16717269 10.1503/cmaj.051528 PMC1459894

[35] Spiller H, Lorenz DJ, Bailey EJ, Dart RC. Epidemiological trends in abuse and misuse of prescription opioids. J Addict Dis 2009;28:130–6. 10.1080/10550880902772431 19340675

[36] Jamison RN, Butler SF, Budman SH, Edwards RR, Wasan AD. Gender differences in risk factors for aberrant prescription opioid use. J Pain 2010;11:312–20. 10.1016/j.jpain.2009.07.016 19944648 PMC2847642

[37] Back SE, Payne RA, Waldrop AE, Smith A, Reeves S, Brady KT. Prescription opioid aberrant behaviors: a pilot study of sex differences. Clin J Pain 2009;25:477–84. 10.1097/AJP.0b013e31819c2c2f 19542794 PMC2771580

[38] Moride Y, Abenhaim L. Evidence of the depletion of susceptibles effect in non-experimental pharmacoepidemiologic research. J Clin Epidemiol 1994;47:731–7. 7722586 10.1016/0895-4356(94)90170-8

[39] Hall AJ, Logan JE, Toblin RL, Kaplan JA, Kraner JC, Bixler D, et al Patterns of abuse among unintentional pharmaceutical overdose fatalities. JAMA 2008;300:2613–20. 10.1001/jama.2008.802 19066381

[40] Centers for Disease Control and Prevention (CDC). Vital Signs: Risk for Overdose from Methadone Used for Pain Relief—United States, 1999–2010. MMWR Morb Mortal Wkly Rep. 2012;61:493–497. 22763888

